# Dung avoidance behavior in Crioula Lanada lambs naturally infected with gastrointestinal nematodes in a rotational pasture system

**DOI:** 10.1590/S1984-29612022012

**Published:** 2022-03-16

**Authors:** Patrizia Ana Bricarello, Leticia Rodrigues Costa, Cibele Longo, Jaqueline Seugling, César Cristiano Basseto, Alessandro Francisco Talamini do Amarante, Maria José Hötzel

**Affiliations:** 1 Laboratório de Parasitologia Animal, Departamento de Zootecnia e Desenvolvimento Rural, Centro de Ciências Agrárias, Universidade Federal de Santa Catarina – UFSC, Florianópolis, SC, Brasil; 2 Laboratório de Etologia Aplicada e Bem-Estar Animal, Departamento de Zootecnia e Desenvolvimento Rural, Centro de Ciências Agrárias, Universidade Federal de Santa Catarina – UFSC, Florianópolis, SC, Brasil; 3 Departamento de Parasitologia, Instituto de Biociências, Universidade Estadual Paulista – UNESP, Botucatu, SP, Brasil

**Keywords:** Voisin Grazing System, parasitism, immune response, nutrition, ethology, sheep, Sistema de Pastoreio Voisin, parasitismo, resposta imune, nutrição, etologia, ovinos

## Abstract

This study aimed to evaluate foraging distance (FD) from the dung, parasitological and physiological factors in 18 Crioula Lanada lambs naturally infected by nematodes with three infection levels (IL) in a Voisin Grazing System. In the pre-experimental phase animal feces collection, deworming, observer training, animal adaptation and dung demarcation were carried out; in the experimental phase, grazing distance, feces, pasture and blood sampling. An initial exploratory analysis was carried out (Kruskal-Wallis test). Fixed predictors were selected with a cumulative logit regression model; an ordinal logistic regression mixed model identified influencing factors of ordinal responses for (i) FD, (ii) infective larvae quantity (L3). Animals approached the dung when the radiation or temperature were more intense (P < 0.05). Paddock entry/exit, IgG and L3 influenced FD over time (P < 0.05). L3, in turn, was influenced by IL, FEC and corpuscular volume (CV). In the High IL group, FD varied between 60-100 cm. Greater L3 and FEC were found in the High and Low IL from the 4th week (P < 0.05). Naturally infected Crioula Lanada lambs increased the distance from the dung, which was not related to IL but to the dynamics of solar radiation and parasitological and immunological factors.

## Resumo

O objetivo deste estudo foi avaliar a distância de forrageamento do bolo fecal (DF), fatores parasitológicos e fisiológicos em 18 cordeiros Crioula Lanada, naturalmente infectados por nematoides com três níveis de infecção (NI) em sistema Pastoreio Racional Voisin (PRV). Na fase pré-experimental, houve coleta de fezes dos animais, vermifugação, treinamento de observadores, adaptação dos animais e demarcação do bolo fecal; na fase experimental, distância, fezes, pastagem e sangue. Um modelo de regressão "logit" cumulativa selecionou preditores fixos; um modelo misto de regressão logística ordinal identificou fatores influenciadores das respostas ordinais para (i) DF (ii) quantidade de larva infectante (L3). Os cordeiros se aproximaram do bolo fecal quando a radiação ou temperatura foram mais intensas (P < 0,05). A entrada e a saída dos piquetes, a ingestão de L3 e IgG influenciaram DF (P < 0,05). L3 foi influenciada por NI, OPG e volume corpuscular. O grupo NI Alta variou a distância entre 60-100 cm. Maior L3 e a FEC foram encontrados nos grupos NI Alta e Baixa a partir da 4ª semana (P < 0,05). Cordeiros Crioula Lanada, naturalmente infectados, aumentaram a DF, sem relação com nível, de infecção, mas com a dinâmica da radiação solar e dos fatores parasitológicos e imunológicos.

## Introduction

Diseases caused by internal and external parasites are one of the most important health restrictions affecting the productivity of ruminants maintained in production systems in tropical regions, where most of the livestock is pasture-based. Loss in industrial sheep production in Brazil and other South American countries is mainly due to the damage caused by gastrointestinal nematodes; parasites can bring about a reduction in physical development and reproductive performance, weight loss, apathy, decrease in voluntary consumption, anemia and mortality (Amarante, 2014).

In ruminants, gastrointestinal parasitic infections are most commonly caused by the ingestion of infective larvae (L3) with food. Herbivores use three strategies to combat parasitic infections while foraging: prevent the infections, resist the parasitism or self-medicate (Hutchings et al., 2006). Grazing animals possess a complex daily decision about foraging, with the objective of maximizing the rate of nutritious ingestion and minimizing the cost, as well as overcoming the risk of parasitic infections (Hutchings et al., 1998). This behavior can be explained, for example, by the cost-benefit decision that the animal makes (the trade-off). The effect can be measured by its change in feeding behavior, where the animal is motivated to select a diet based on the damage caused by the parasitic infection. The trade-off may only be visible one week after infection (Hutchings et al., 2001a). The more attractive pastures, which usually possess greater nutritional value, are typically located in places having a large amount of L3 contamination, thus producing the conflict of choice between better nutrition and fecal avoidance behavior (Hutchings et al., 1999).

It is believed that when avoiding dung, the animal limits its contact with pathogenic agents that are transmitted fecal-orally, consequently reducing the risk of infection (Hutchings et al., 2001b). Feces serve as a reservoir of helminth larvae in the pasture, and depending on the environmental and climatic conditions, this material may be conserved for up to sixteen weeks (Rocha et al., 2014). Food selection helps to reduce the parasite burden of the animal (Stear et al., 1995), an event which has been observed in domestic animals and in wild ungulates.

Some phenotypic markers such as the number of eggs per gram of feces, packed cell volume, number of circulating eosinophil and antibody levels have been associated with parasite resistance (Bricarello et al., 2007). Sheep resistant to nematodes have been selected for over the past several decades and this has resulted in animals with different immunological and behavioral characteristics (Hutchings et al., 2007). The distance of foraging behavior from dung could be related to the level of natural resistance in sheep and behavioral adaptations may be part of the host's means of controlling gastrointestinal parasites.

Crioula Lanada is a rustic sheep breed formed by the crossbreeding of animals with Portuguese and Spanish ancestry taken to Brazil during the 18th century colonial period. The survival of the animals that adapted to the terrain, climate, and local difficulties of South America originated a very rustic dual-purpose breed that has a distinctive naturally-colored fleece used in handicraft, clothing, carpet, blanket, and meat production (Moreira et al., 2021). Regarding resistance to gastrointestinal nematodes, Crioula Lanada lambs have greater resistance to infection by *H. contortus* (Bricarello et al., 2002; Bricarello et al., 2004) and ewes have moderate nematodes parasitic infections during parturition and lactation (Pereira et al., 2020).

The magnitude and occurrence of parasitism outbreaks are influenced by the immunity and the feeding behavior of the host and their interactions (Séo et al., 2015). Understanding the physiological and behavioral defense strategies may help develop new approaches to sheep nematode control.

Voisin Grazing System (VGS) is a pasture-based rotational system used to manage the soil-plant-animal relationship. The use of the paddocks is determined by adequate plant rest period, in which each paddock is used at a high stocking rate for a short period of time (Machado, 2004). This system aims to improve the process that transforms solar energy into chemical energy in the pasture, without the external interference of synthetic elements (e.g. agrochemical fertilizers) and soil friction (e.g. plowing) (Balcão et al., 2017). Beef cattle in the VGS most affected by parasites grazed farther from dung, demonstrating the relationship between behavior and immune system (Séo et al., 2015).The avoidance behavior is probably an animal strategy to prevent parasitic infections.

This study aimed to evaluate the grazing behaviour, parasitological and physiological factors in Crioula Lanada lambs naturally infected by gastrointestinal nematodes with three infection levels and maintained in a rotational grazing system, correlating these data with climatic factors.

## Material and Methods

### Experiment site and climate data

The experiment was carried out at the Núcleo de Pesquisa e Extensão em Agroecologia da Fazenda Experimental Ressacada of the Universidade Federal de Santa Catarina in Florianópolis, Santa Catarina, Brazil (27^o^41’06.28”S; 48^o^32’38.81”W) (altitude: 2-4 m above sea level). The climate is wet subtropical of *Cfa* type according to Köppen-Geiger climate classification and, geologically, formed by marine sedimentation processes, with the soil classified as hydromorphic quartzarenic neosol.

Environmental temperature, humidity, solar radiation and rainfall data during the period of the experiment were provided by a meteorological station in Floripa Airport, located 1.3 km away from the experiment site in the south region of Florianopolis, SC, Brazil.

### Experimental design, climate data and animals

The experiment was carried out in spring/summer (October to December 2015). A total of 20 paddocks were used (mean area: 208 m^2^) that consisted of pasture composed of *Brachiaria* sp, *Cynodon dactylon*, *Trifolium repens*, *Paspalum notatum*, *Setaria sphacelata*, *Desmodium tortuosum*, *Trifolium pratense* and *Axonopus jesuiticus*.

The experiment occurred in two phases: a pre-experimental (October) and an experimental phase (November 23rd to December 23rd). The pre-experimental phase consisted of animal feces collection, deworming, observer training, adaptation of animals to observer contact and placement of stakes (height: 1 m; width: 4.2 mm) in the paddock to demarcate dung. In order to decrease interfering with animal behavior, stakes were placed in the entire paddock, some demarcating feces and others in randomly-chosen areas. The tip of the stakes had one color for those demarcating the dung and another for those set randomly. The dung came from the natural on-site defecation. When the lambs entered the paddock, dung that from the last time the same animals had been there was marked, so that this dung was at least 30 days old.

In the experimental phase (November 23rd to December 23rd), behavioral analyses, animal feces sampling, blood collection, weighing of the animals, and pasture collection and analysis were carried out. The dung was demarcated 24 hours after the animals exited the paddock.

Weekly blood and feces sampling, weighing of the animals and determination of the body condition score happened on Wednesdays, starting at 07h30min. Feces collections were carried out individually. The feces were removed directly from the rectum, stored in individual bags and identified. These samples were used for fecal egg counts (FEC) and coprocultures. Blood samples were taken using jugular venipuncture to evaluate corpuscular volume, total plasma protein, blood eosinophil count and IgG antibody level and these methodologies are described in detail in the section Hematological and immunological analyses.

A total of 18 (10 females and eight males) 4-month-old Crioula Lanada lambs were used. The animals were identified individually with a colored ribbon around the neck. The project was approved by the Ethics Committee on Animal Use of the Universidade Federal de Santa Catarina (CEUA/UFSC) under the protocol N^o^PP00929.

Precipitation and relative humidity did not vary during the experiment. Temperature varied 7^o^ C (20 to 27^o^ C). Mean radiation was 1,055 kj/m^2^, varying greatly between 411 and 1,998 kj/m^2^. Mean humidity was high at 84% ± 5.7.

### Behavioral analyses: a foraging distance from the dung

Every measurement taken at the moment of the behavioral analysis, which constituted a foraging distance from the dung less than 1 m between foraging and dung, was duly noted. Forage samples consumed less than 1 m from the dung were collected to evaluate the quantity of L3/kg DM (Dry Matter) ingestion (Hutchings et al., 2007; Séo et al., 2015).

The observations aimed to evaluate the response of the animals to a new parasite infection after anthelmintic treatment. The grazing behavior when entering the paddock under the VGS and the grazing behavior during the stay were evaluated. The observations were carried out on Mondays, Tuesdays, Thursdays and Fridays from 08h00 min to 10h15 min. Each animal was observed individually for five minutes, with every animal of each observer observed sequentially, according to its marking (repeated three times). The animals entered the paddock at 07h30min and allowed 30 min for adaptation to and recognition of the paddock, with the behavioral evaluations and grass sampling (when foraging occurred less than 1 m from the dung) starting soon after. Pasture was collected, with the help of a 20x20 cm square, at the site where the animal was grazing, and stored for parasitological analysis. Animal behavior was evaluated after 24 hours in the paddock. Grass collections were also carried out as previously described.

The observations were carried out by two researchers. One observer, who had no knowledge of the degree of infection of the animal, always accompanied the same group of 9 animals, which was randomly assigned (Altmann, 1974; Lehner, 1996). A total of 3 individual observations (snapshots) of each animal were made every 5 minutes. Distance from the dung while grazing was recorded after each observation. Weekly behavioral observations were made for 5 weeks, along with grass samplings on Mondays, Tuesdays, Thursdays and Fridays. The observations were made on Mondays and Thursdays, when the animals entered the paddock, and on Tuesdays and Fridays, after a 24-hour stay.

### Parasitological measurements

#### Fecal egg counts and fecal larval culture

Fecal egg counts (FEC) and larval cultures were carried out using the modified technique by Ueno & Gonçalves (1998). The infective larvae (L3) were identified according to Keith (1953). The coprocultures were done in the third and fourth weeks of the experiment.

FEC were done weekly for each animal, starting at 21 days after birth, for 16 weeks (pre-experimental phase). The lambs were divided into three groups of six, according to infection level: Low Infection (FEC < 500), Medium Infection (FEC 501-1000) and High Infection (FEC > 1000). Every lamb was treated with albendazole-based anthelmintics (10 mg/kg, Ibazole®, Ibasa), associated with levamisole hydrochloride (10 mg/kg, Ripercol® L 150 F, Fort Dodge), for 3 consecutive days, 7 days before starting the observations. This combination of anthelmintics promoted a reduction in the general FEC mean from 502.28 to 5.55 on the first day of the experimental phase. The lambs stayed in the paddocks under the VGS, with the one rotation stipulated by the system, staying 24 to 36 h in each paddock. After this period, they were taken to a paddock located in a restful area (not necessarily adjacent). Every paddock had constant access to water, mineral supplements and shade. The lambs received 300g/animal/day of the commercially-available sheep supplement Presence® (14% crude protein).

#### Recuperation of infective larvae from forage

Pasture samples were collected before the animals entered the paddock, in order to estimate its parasitic contamination (Séo et al., 2015). The samples were packed in plastic bags that identified the animal, paddock, day, sampling and distance from the dung.

Estimation of L3 ingestion was done by collecting pasture using a 20cm-radius hoop at the exact grazing site at the moment of animal bite (Hutchings et al., 2007; Séo et al., 2015). The pasture in the hoop area was cut at 3 cm above soil and the material was stored in plastic bags and transported to the lab for L3 recovery (Rocha et al., 2007).

The procedure to recuperate larvae in the grass was carried out according to Niezen et al., (1998). The grass samples were briefly immersed in buckets containing 4 liters of water for 4 hours. They were later transferred to another bucket containing 4 liters of water for 3 more hours, remaining submerged for a total of 7 hours. The samples were subsequently removed and weighed, packed in dry paper bags, put in a forced air ventilation oven at 60 °C for 72 hours, and weighed again to verify the dry matter content. The buckets containing the water with the larvae remained at rest for 14 hours. The supernatant was later removed and the sediment transferred into a sedimentation cup, remaining at rest for 24 hours. Once again, the supernatant was removed and the sediment packed into a conical tube, which was refrigerated until the moment of the reading. The supernatant was removed and only 1 mL of the sediment was used to quantify and classify the infective gastrointestinal parasites. The L3 values found were expressed as the quantity of L3 per kilogram of dry matter (L3/kg/DM).

#### Hematological and immunological analyses

Corpuscular volume (CV) was determined using the microhematocrit method and blood eosinophil counts (EOS) were performed in a Neubauer chamber after staining with Carpentier solution (Dawkins et al., 1989), expressed as the number of cells per microliter of blood. Total plasma protein levels (TPP) were estimated using a refractometer (Atago SPR-N Refractometer). An aliquot of plasma was stored in polyethylene Eppendorff flasks and maintained at 20^o^ C for later evaluation of IgG immunoglobulin.

The protocol used for IgG evaluation was described by (Bassetto et al., 2009), with some modifications. The evaluation was carried out from the production of L3 antigens of *H. contortus*, previously described by Bassetto et al. (2009). The process was performed using polystyrene microplates containing 96 antigen-sensitized wells (F96 MicroWell plate, Maxisorp®, NUNC, USA) (100µl of antigens (2 µg/mL) diluted in carbonate buffer (pH 9.6)). The plates were incubated overnight a 4^o^ C. Every following incubation was conducted for 1 hour at 37^o^ C, using 100 µl of reagent in each well. The plates were washed three times between each procedure with ultrapure water (EASYpure II UV, Barnstead, USA) containing 0.05% Tween™ 20 (ProPure® -- Amresco, USA). After sensitization, the plates were blocked with 0.1% gelatin (Amresco, USA) and 0.05% Tween™ 20 in PBS pH 7.2 (PBS-GT). The serum samples were diluted in PBS-GT (1:500) and applied in duplicate. The plates were then incubated with a rabbit anti-sheep (IgG) secondary antibody, conjugated with peroxidase, diluted 1:40000 (A130-101P, Bethyl Laboratories, Inc., USA). Lastly, a solution with the substrate OPD (1,2-phenylenediamine dihydrochloride, Dako, Denmark) was added to each well. The enzymatic reaction was carried out at room temperature, in the dark, for 15 minutes and stopped using a 5% solution of sulfuric acid. The plates were read soon after, using an automatic ELISA reader (Biotrak II, Amersham-Biosciences, UK) at 492 nm. The negative control came from an infection-free sentinel animal, as described by Santos et al. (2014). The positive control came from an animal artificially infected by *H. contortus* and *T. colubriformis* every three days for 84 days. Results for IgG were expressed as the percentage of the optical density value of the positive-reference serum.

### Statistical analysis

An exploratory observational design with repeated measures over time was used. The main response variable was the foraging distance (cm) from the dung as an evaluation measure of grazing behavior and quantity of L3. Foraging distance from the dung (distance), parasitological (estimate of larval ingestion using infective larva “L3” level and FEC), hematological (CV, TPP, EOS), immunological (IgG) and weight of the lambs were submitted to the Shapiro-Wilk test for normality (P < 0.05), which confirmed abnormal distributions, except for weight. Due to the high instability of the data collected, the log (log10 (x+1)) or Box-Cox transformation of data did not result in the normalization of the responses.

An initial exploratory analysis was carried out through the non-parametric Kruskal-Wallis test for a univariate evaluation of predictors (“observation” time, entry/exit, eosinophils, protein and IgG etc) of distance and L3 responses. The probability of error < 5% (P < 0.05) was accepted as having a significant effect on the responses. Probability of error values less than 0.001 were expressed P < 0.001.

The Pearson chi-square test, the McNemar test (in the case of association between two factors with two categories each) (i.e. 2x2 contingency tables), or the Fisher test (when the quantity in the table cell was fewer than 5 units) was used in the frequency analyses of the events of the categorical variables.

In order to pre-select fixed predictors for a mixed model, the cumulative logit regression model (also known as the proportional-odds model) was used (McCullagh, 1980) for ordinal responses for (i) distance between foraging and dung and (ii) quantity of infective L3 larva, based on the vglm function from the VGAM package (Yee, 2015) of the R computational program (R Development Core Team, 2011) That was obtained by performing sequential regressions dropping one variable at a time of the full model. The full model was also compared with the null model, i.e. in the model with the intercept and random variables only.

Finally, in order to identify possible influencing factors, an ordinal logistic regression mixed model was carried out also for the ordinal responses distance and L3. The clmm function from the ordinal package (Christensen, 2015) of the R computational program was applied (R Development Core Team, 2011). The variables and their respective response classes are described in Table 1 and were categorized based on their quartiles. Animal, observations, mean temperature and radiation were chosen as random effects for both models. Comparison of the sequential models with the full model was done using the Deviance criterion (Residual Deviance), the Akaike information criterion (AIC) and the maximum log-likelihood function (LR). When the sequential model presented a higher value of Deviance and AIC criteria and lower LR, which is equivalent to maximizing “negative” L, it demonstrated the importance of the dropped variable and thus it was selected. The likelihood test was also used for the mixed model through ANOVA (R Program), comparing the full model with the null and the sequential model (probability of error 0.05).

**Table 1:** Response classes of parasitological variables (larvae with infection level “L3”, FEC), hematological (CV, PPT, EOS), immunological (IgG) of distance from the dung.

	Response Classes				
	1	2	3	4	5
EOS	< 250	250-800	> 800		
PCV	< 30	30-34	> 34		
PPT	< 7	7-7,3	7,3-7,9	>7,9	
IgG	< 45	> 46			
L3	0	0-80	> 80		
Distance	Very close	Close	Far	Vary far	Without collection
Distance	0-15 cm	16-30 cm	31-60 cm	61-99 cm	> 100 cm

## Results

### Climatic influence on foraging distance and L3 ingestion

Only radiation and mean air temperature had an effect (P < 0.05) on foraging distance and ingestion of L3/kg DM and were selected as random effects for the mixed models.

**Figure 1:**
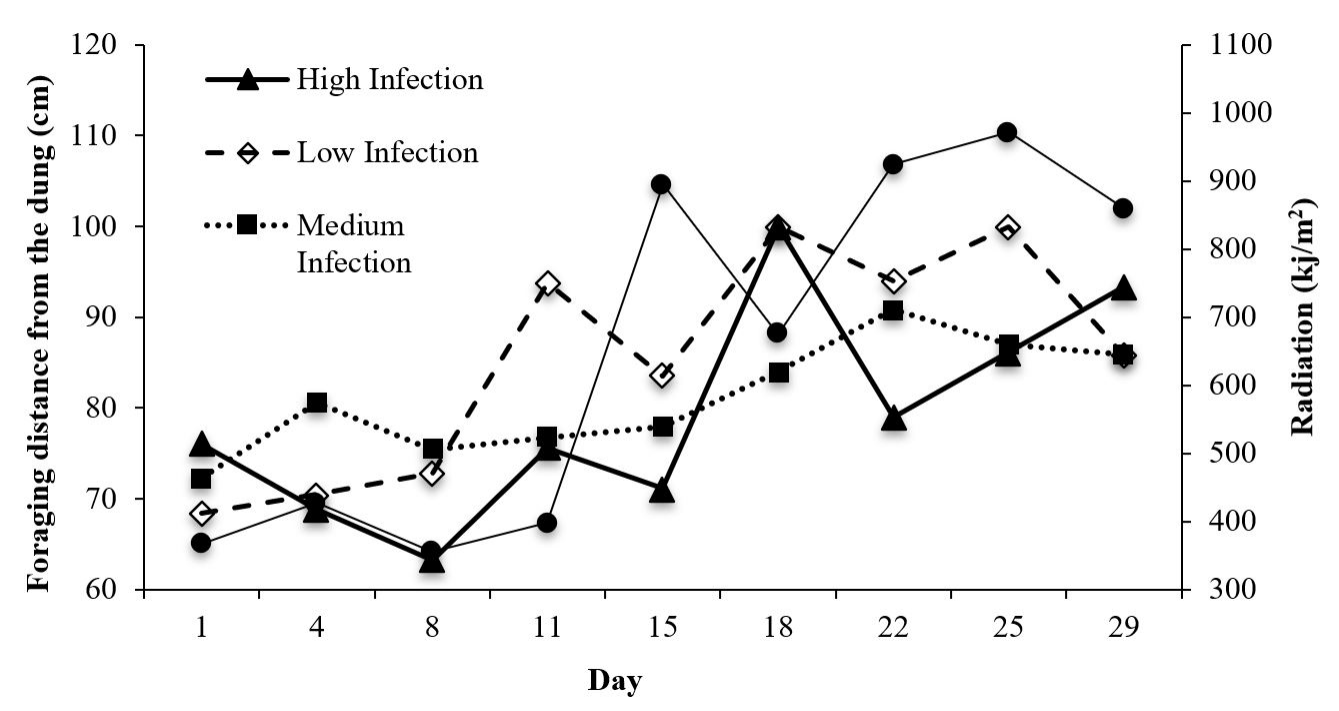
Foraging distance from the dung (cm) and solar radiation (kj/m^2^) in the Low, Medium and High Infection groups of Crioula Lanada lambs during experimental days.

**Figure 2:**
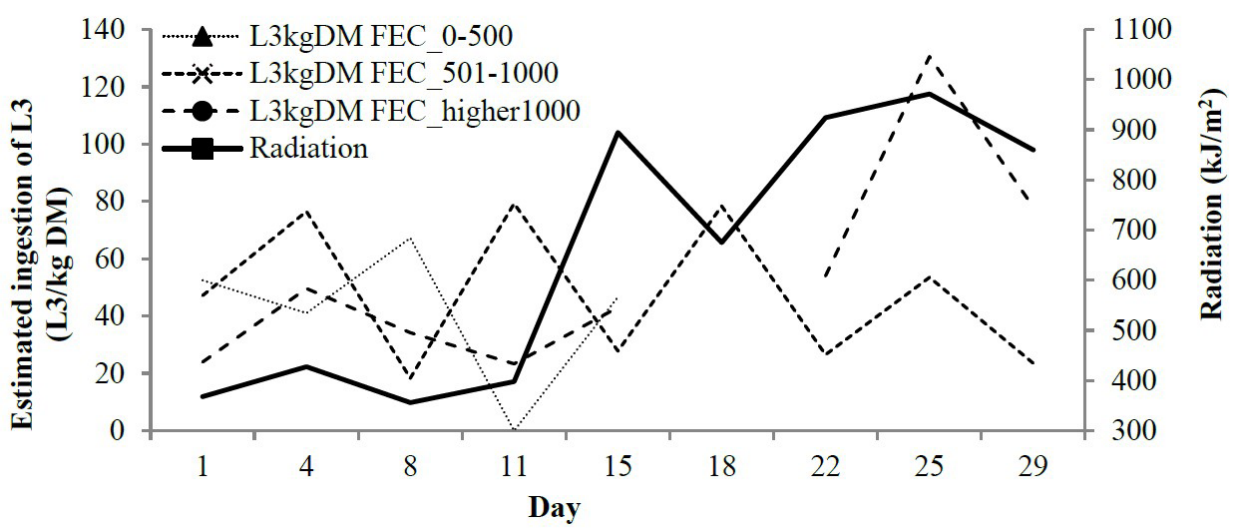
Foraging distance from the dung in cm measured when lambs of the Low, Medium and High Infection groups of Crioula Lanada lambs entered the paddock lambs during experimental days.

The animals approached the dung when the radiation was more intense (Figure 1), except for the Medium Infection group, which always stayed away from the dung from the 15^th^ day. L3 ingestion increased when the radiation was more intense for the High and Low Infection groups (Figure 2).

### Behavioral foraging: distance from the dung over time in VGS

Initially, a gradual increase in foraging occurrences farther than 100 cm over the weeks of the experiment was observed (P < 0.01). The proportion of foraging occurrences farther than 100 cm was 46, 57, 72, 84 and 86% from the 1^st^ to the 5^th^ week, respectively (P < 0.001). In the 1^st^ and 2^nd^ weeks, on average, 36% of the foraging occurred up to 60 cm from the dung; whereas in weeks 3, 4 and 5, only 17, 15 and 14% (P < 0.0001) of the animals, respectively, were observed near the dung (Figure 1).

**Figure 3:**
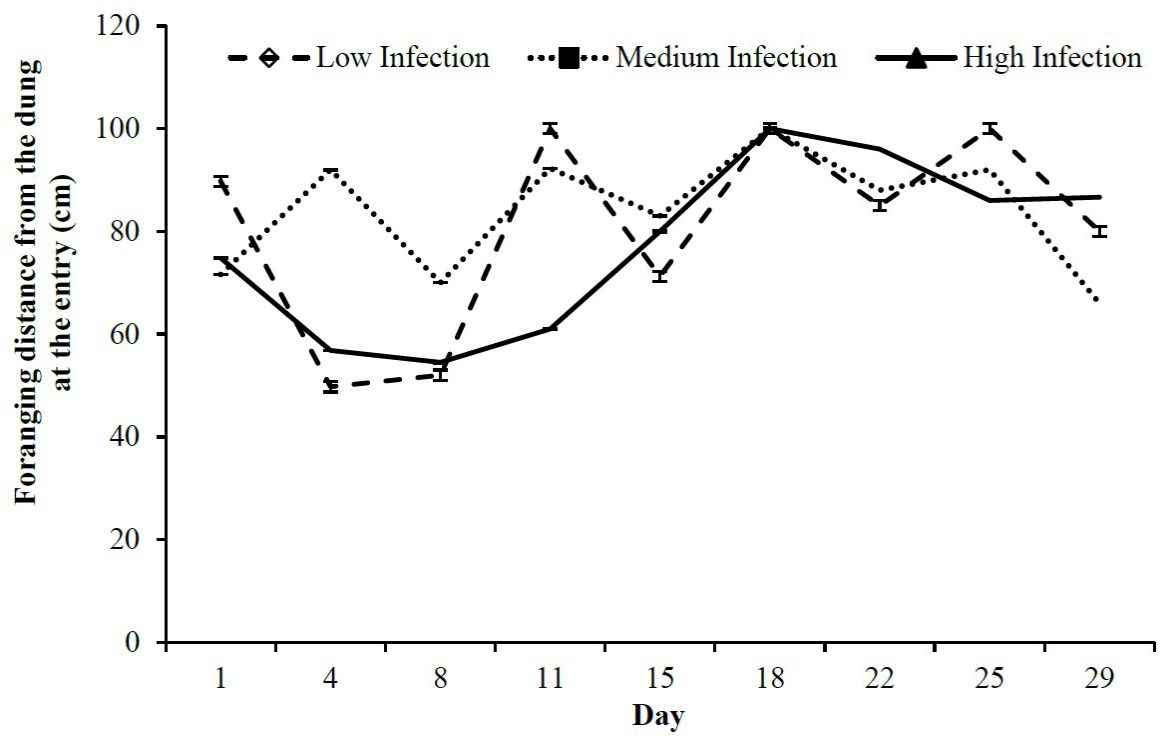
Foraging distance from the dung in cm measured when lambs of the Low, Medium and High Infection groups of Crioula Lanada lambs exited the paddock lambs during experimental days.

**Figure 4:**
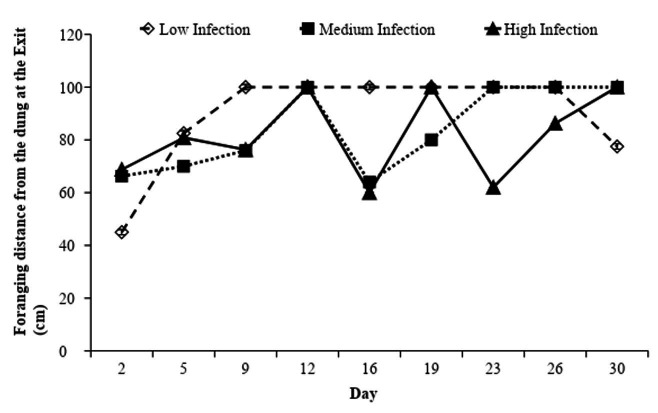
Fecal egg counts (FEC) occurring in the Low, Medium and High Infection groups of Crioula Lanada lambs during experimental days.

There was no difference in foraging distance over time between infection levels (P > 0.05). In general, most foraging events (67.3%) occurred farther than 100 cm from the dung, independent of infection level (P = 0.16). When the animals entered the paddock (Figure 3), lambs of all groups kept away at least 50 cm from the dung from the 15^th^ day. When they exited the paddock (Figure 4), the lambs of the Low Infection group had maintained distances above 100 cm since the start of the experiment, while the High Infection group had varied its foraging distance between 60 and 100 cm. The intermediate group had occurrences between 68 and 100 cm only up to the 24^th^ day, when, on average, the animals grazed 100 cm from the dung.

### Factors influencing foraging distance over time in VGS

The pre-selected fixed predictors of the distancing from the dung were estimated by the logito cumulative regression model for ordinal responses (vglm model: ordered(distance) ~ infection level + sex + entry/exit + L3kgms + FEC + CV + TPP + EOS + IgG). These vglm sequential models model were compared with the full and null model. As a result, *infection level, entry/exit, ingested L3, FEC* and *IgG* were selected as possible influencing factors (Table 2) for the distance response and used as fixed predictors in the ordinal logistic regression mixed model (clmm) .

**Table 2:** Evaluation parameters for the cumulative logit regression analysis (vglm) for foraging distance in the full, null and sequentials models.

Models	Residual deviance	log-Likelihood	AIC	df%
Full	222	-111	248	257
Null	599	-299	608	1108
*Sequential models^+^*				
Infection level	224	-112	246	259
sex	222	-111	246	258
entry/exit	228	-114	252	258
L3/kg DM	568	-284	594	1095
FEC	224	-112	248	258
CV	222	-111	246	258
TPP	222	-111	246	258
EOS	222	-111	246	258
IgG	224	-112	248	258

Full model = vglm(ordered distance) ~ infection level + sex + entry/exit + L3kgms + FEC + CV+ TPP + EOS + IgG, family = cumulative (parallel = T), data = data's name); Null model: ordered distance ~ 1, family=cumulative(parallel=T), data= data's name);

+ sequential regressions dropping following variable at a time of the full model; entry/exit = data from the entrance or exit of the paddock; L3 = infective larvae; FEC = fecal egg counts; CV = corpuscular volume; TPP = total plama protein; EOS = eosinophil counts; IgG = antibody level;

^%^ degree of freedom.

The five mentioned pre-selected variables were introduced in the ordinal logistic regression mixed (clmm) for the ordinal response distance (clmm model: ordered Distance = infection level+ entry/exit + L3kgms + FEC + IgG + (animal + observations + mean temperature + radiation) and the sequential clmm models compared with the full and null model (Table 3).

**Table 3:** Evaluation parameters for the ordinal logistic regression mixed (clmm) for the ordinal response foraging distance in the full, null and sequentials models

Models	log-Likelihood	AIC	Anova*
Full	-111	248	
Null	-289	594	nd#
*Sequential models* +			
Infection level	-112	245	0.5801
entry/exit	-113	250	0.0558
L3kg/DM	-282	590	nd
FEC	-112	248	0.2681
IgG	-113	249	nd

Full model: clmm(ordered distance) ~ infection level+ entry/exit + L3kgms + FEC + IgG + (1|animal) + (1|observacao) + (1|tempmedia) + (1|radiacao), na.action=na.omit,data=data's name); Null model: clmm(ordered distance) ~ 1 + (1|animal) + (1|observacao) + (1|tempmedia) + (1|radiacao), na.action=na.omit,data=data's name);

+ sequential regressions dropping following variable at a time of the full model; entry/exit = data from the entrance or exit of the paddock; L3 = infective larvae; FEC = fecal egg counts; CV = corpuscular volume; TPP = total plama protein; EOS = eosinophil counts; IgG = antibody level;

* anova method = likelihood ratio test (comparison between full model and null model or full modell and sequential models);

# nd = not determined.

There was a reduction in the AIC value from 594 to 248 and maximization of the likelihood ratio (LR) from -289 to -111 when these variables were introduced in the null model.

When entry/exit, L3 and IgG were removed individually from the full model, AIC and LR were as high (568 and -284, respectively) as in the null model (599 and -299, respectively), indicating that the moment of entrance and exit, the quantity of larvae ingested (close to a mouthful) in the pasture, and immunological system were associated with foraging distance from the dung.

The β coefficient for the L3 predictor was negatively significant (-0.98; P = 0.0013) in every sequential model and the β coefficient for IgG was always positively significant (1.02; P < 0.05) in every model evaluated. As regards to the variable L3, starting with the shortest distance category, i.e. category 1 (very close) up to category 4 (very far) as described in Table 1, there was a reduction of 0.98 larval counting units (P = 0.0013), i.e., that the quantity of L3 removed from the pasture was inversely related to the distance category. On the other hand, there was an increase of 1.02 IgG units with the increase in the foraging distance from the dung, which mostly occurred with the low infection level.

The likelihood ratio tests (ANOVA) between the full model and the sequential models tested fixed variables were not significant (P > 0.05) for infection level and FEC showing that they were not directly associated with foraging distance. In summary, the regression analyses showed that only *entry/exit, IgG* and especially *L3 ingestion* were the factors influencing foraging distance over time.

### Estimated ingestion and recuperation of L3 larvae over time in VGS

The estimated ingestion of L3 varied according to foraging distance and weeks of experiment (P < 0.05). In general, the estimate of the quantity of L3 ingested by the animals was lower the farther foraging was from the dung. The animals that grazed more than 40 cm from the dung presented mean ingestion values of 0 L3/kg DM in contrast to the estimated ingestion of 55 L3/kg DM for the animals that grazed between 15 and 40 cm from the dung and 72 L3/kg DM for the animals that grazed less than 15 cm from the dung (Figure 2).

Greater L3 ingestion was observed in the High and Low Infection groups from the 4^th^ week of the experiment. There was a reduction in the mean values of L3 ingestion in the Medium Infection group over the period of the experiment. No significant difference was detected in the quantity of L3/kg DM ingested between entering and exiting the paddocks (P = 0.32), which was confirmed in the regression analyses.

In the pre-experimental phase, when the animals entered the paddocks, the pasture was contaminated with L3 of the following nematode genera (means from the entire period): *Haemonchus* (41%), *Cooperia* (35%), *Trichostrongylus* (18%) and *Oesophagostomum* (6%).

The fecal cultures contained *Haemonchus* (66%), *Cooperia* (18%) and *Trichostrongylus* (16%) in the third week of the experiment and *Haemonchus* (71%), *Cooperia* (26%) and *Trichostrongylus* (3%) in the fourth week.

*Haemonchus* occurred in 46% of the samples of pasture that the animals had grazed less than 60 cm from the dung. This percentage fell to 22% (P < 0.05) in samples that the animals had grazed between 60 and 100 cm from the dung.

### Factors influencing ingestion of L3 larvae from the pasture over time in VGS

The cumulative logit regression analysis for L3 as a response variable (vglm model = ordered L3 ~ infection level + sex + entry/exit + FEC + CV + TPP + EOS+ IgG) pre-selected *infection level, FEC, CV* and *eosinophils* as possible influencing factors (Table 4) for L3 ingestion from pasture, discarding sex, entry/exit, TPP and IgG. Those selected variables were then tested as fixed variables in the cumulative link mixed model in ordinal response (clmm), inserting animals, observation temperature and radiation as random parameters.

**Table 4:** Evaluation parameters for the cumulative logit regression analysis (vglm) for ordinal response L3 ingestion in the full, null and sequentials models.

Models	Residual deviance	log-Likelihood	AIC	df%
Full	171	-85	193	169
Null	195	-97.6	199	180
*Sequential models* +				
Infection level	182	-91	200	171
sex	171	-85	191	170
entry/exit	171	-85	191	170
FEC	176	-88	196	170
CV	172	-86	192	170
TPP	171	-85	191	170
EOS	172	-86	192	170
IgG	170	-85	191	170

Full model: vglm(ordered(L3) ~ infection level + sex + entry/exit + FEC + CV+ TPP + EOS + IgG, family=cumulative(parallel=T), na.action=na.omit,data=data's name); Null model: vglm(ordered(L3) ~ 1,family=cumulative(parallel=T), na.action = na.omit, data = data's name);

+ sequential regressions dropping following variable at a time of the full model; entry/exit = data from the entrance or exit of the paddock; FEC = fecal egg counts; CV = corpuscular volume; TPP = total plama protein; EOS = eosinophil counts; IgG = antibody level;

^%^ degree of freedom.

When introducing these four predictors into the null model, there was a significant reduction in AIC from 204 to 195 and maximization of the LR from -96 to -85, demonstrating the better fit of the full model (Table 5). Likewise, when comparing the full model with the sequential models, the likelihood ratio test (ANOVA) was significant (P < 0.05) for *infection level* and *FEC*. Minimization of LK and higher AIC occurred when *ordered CV* was dropped out of the full model, indicating its influence in L3 ingestion.

**Table 5:** Evaluation parameters for the ordinal logistic regression mixed (clmm) for the ordinal response L3 ingestion in the full, null and sequentials models.

Models	log-likelihood	AIC	Anova*
Full	-85	195	
Null	-96	204	nd#
*Sequential models* +			
Infection level	-89	199	0.0202
FEC	-88	198	0.0197
Ordered CV	-89	199	nd
EOS	-86	194	0.3413

Full model: clmm(ordered L3) ~ infection level + FEC+ ordered CV + EOS + (1|animal) + (1|observacao) + (1|tempmedia) + (1|radiacao), na.action = na.omit, data = data's name); Null model: clmm(ordered L3) ~ 1 + (1|animalF) + (1|observacao) + (1|tempmedia) + (1|radiacao), na.action=na.omit,data=data's name);

+ sequential regressions dropping following variable at a time of the full model;

entry/exit = data from the entrance or exit of the paddock; FEC = fecal egg counts; CV = corpuscular volume; TPP = total plama protein; EOS = eosinophil counts; IgG = antibody level;

* anova method = likelihood ratio test (comparison between full model and null model or full modell and sequential models);

# nd = not determined.

Nevertheless, AIC values of the model without eosinophils (194) had an increase of 1 units compared to the full model but non significance in the anova test (P > 0.05), which indicates that it was not a good predictor to L3 estimated ingestion.

In summary, *infection level, FEC* and *CV* were the factors influencing ingestion of L3 larvae from the pasture according to the regression analysis.

### Parasitological, hematological and immunological parameters as regards infection level over time

**Figure 5:**
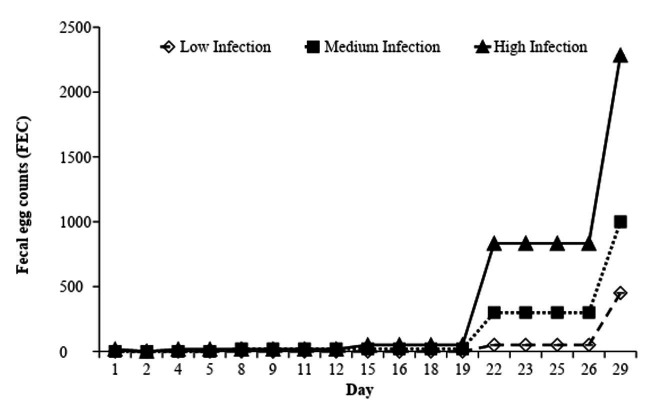
Blood eosinophil count (cells per microliter of blood) in the Low, Medium and High Infection groups of Crioula Lanada lambs during experimental days.

There was an interaction between FEC and infection levels (P < 0.05) in the 4^th^ and the 5^th^ weeks of the experiment, when the FEC values of the High Infection group were significantly higher, followed by the Medium Infection group and the Low Infection group (Figure 5). The first eggs occurred in the samples collected on day 19. The egg elimination peak occurred from day 26 for the three infection levels.

**Figure 6:**
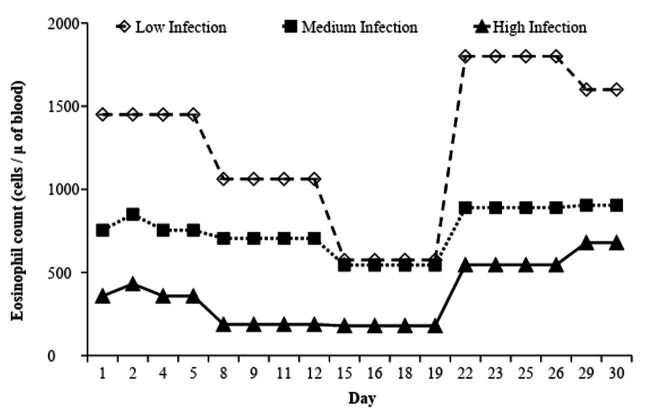
Corpuscular volume (CV %) in the Low, Medium and High Infection groups of Crioula Lanada lambs during experimental days.

When the FEC was at its highest, there was also an elevation in the blood eosinophil counts in every group (Figure 6). Beginning on the 19^th^ day, there was an increase in the eosinophil count, with the highest values occurring in the Low Infection group, followed by the Medium Infection group and the High Infection group (P < 0.05)

**Figure 7:**
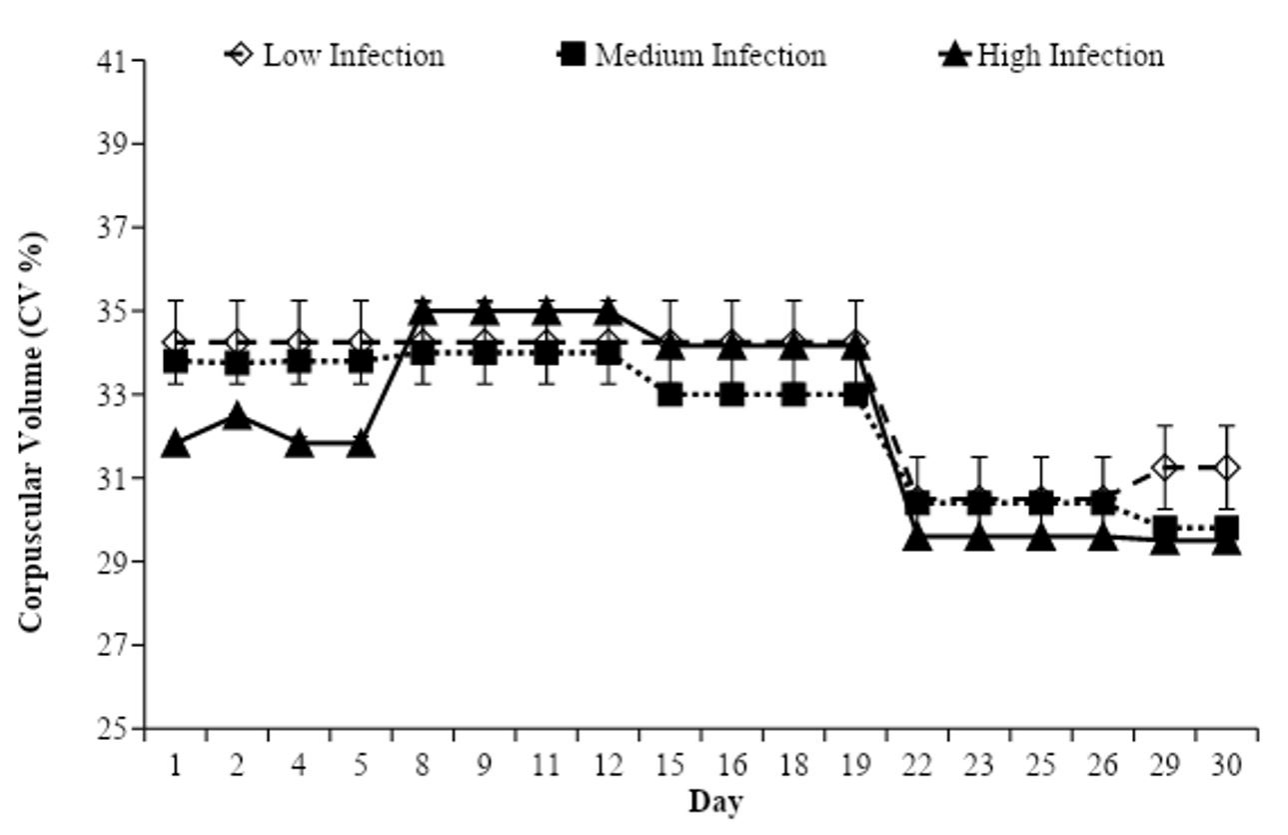
IgG evaluation expressed in percentage of Optical Density (OD %) value of the positive-reference serum in the Low, Medium and High Infection groups of Crioula Lanada lambs during experimental days.

There was an interaction between weeks and infection levels in the CV, especially between the 2^nd^ and the 4^th^ weeks (P < 0.05). The High Infection group had an increase of 10% on the 5^th^ day, remaining high until day 22. In general, there was a fall in CV on the 22^nd^ day for every group (Figure 7).

During the experiment, the TPP dynamics presented significant differences between the Low Infection group and the Medium Infection group in the 5^th^ week (P < 0.05). However, the TPP values of every group were considered normal for the ovine species and, therefore, not characterized as hypoproteinemia.

**Figure 8:**
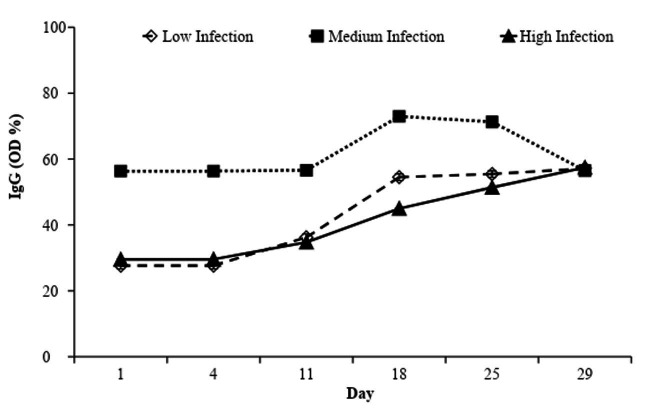
Estimated ingestion of L3 per kilogram of dry matter (L3/kg/DM) and solar radiation (kj/m^2^) in the Low, Medium and High Infection groups of Crioula Lanada lambs during experimental days.

The groups had different IgG concentrations (P < 0.05) during the experiment. An elevation of IgG values occurred in the High and Medium Infection groups from the 4^th^ day. In the High Infection group, the values continuously increased until the end of the experiment; in the Medium Infection group, there was a reduction in IgG values from 77.4% to 64.1% from the 25^th^ day. IgG values increased in the Low Infection group from day 11, stabilizing at 55.5% on day 18 (Figure 8).

## Discussion

Lambs from all groups increasingly stayed away from the dung during foraging over time, independent of their parasitic states. Immunological alterations capable of moderating the dung avoidance behavior were more important than the parasitic condition of the animals. The immune response of the host is an important regulator of the seasonal transmission dynamics of helminths (Fox et al., 2013). The group of animals that presented the lowest immune response performance (characterized by the lower eosinophils and IgG responses) and highest fecal egg counts almost always foraged closer to the dung, ingesting larger quantities of L3.

The animal is constantly making trade-offs between meeting its nutritional needs (more food and of better quality food and maintaining itself healthy (Hutchings et al., 2006). All the animals behaved in a way to minimize the ingestion of infective larvae; however, they differed in the way they attained this objective. When searching for nutritionally better pastures, occasionally moving closer to the dung, the lambs from the High Infection group sought to increase their immunological defenses; in contrast, the lambs from the Low Infection group did not need to take risks. In animals under grazing conditions behavior may influence the time and the intensity of the parasitic outbreaks (Fox et al., 2013). The trade-off theory may explain this cost/benefit relationship behavior, essential in a particular risky situation (e.g. helminth infections) (Hutchings et al., 2001a). The more attractive pastures, which usually possess greater nutritional value, are typically located in places that have greater L3 contamination, thus producing this trade-off between better nutrition and dung avoidance behavior (Hutchings et al., 1999).

The increase in IgG values in the High Infection group was insufficient to maintain the reduction in the FEC given that the Low Infection group. The Low Infection group strategically kept away from the dung, which explains the laxity of the immune response, since there was no ingestion of L3 in this group that was large enough to activate the immune system. The degree of contamination of the pasture may interfere in the grazing behavior of the animal and affect its physiological state, due to the damage caused by the parasite in the intestinal tract of the animal (Coop et al., 1982). Herbivores do not detect parasites in the pasture; however, they use fecal odor as a way to detect parasites in the environment (Dohi et al., 1991). When managed under Voisin Grazing System, the animals spend a short period in the paddocks and, therefore, modifications in foraging behavior at the start (entry) and at the end (exit) of occupation are expected (Séo et al., 2015). The animals tend to graze far from the dung due to accumulation of feces along the period, which explains the greater number of grazing occurrences near the dung at entering than at exiting the paddock. Thus, the L3 ingestion alternation, over the weeks of the experiment, led to the withdrawal of all the animals from the dung; however, this alternation favored risk-taking in the High Infection group. This behavior was later affected by the immunological state and the distribution of the parasites in the environment, i.e. by the contamination of the paddocks by infective larvae when the lambs entered and exited the paddocks. However, in the first weeks all the animals risked foraging closer to the feces. The behavioral changes in the Low Infection group started on the 9^th^ day, and in the High and Medium Infection animals from the 16^th^ and 18^th^ day, demonstrating a behavioral change during the prepatent period of the infection.

Climatic conditions may also interfere in grazing behavior. Our results showed that the increase in radiation increased foraging distance from the dung. This withdrawal of the lambs from the dung may occur when radiation affects a pasture with little forage cover, making the repellent fecal odor stronger (Dohi et al., 1991). On the other hand, high radiation may, with the weather, also provoke desiccation of the L3, which favors the animals (Rocha et al., 2007). The region where the study was carried out is a tropical coastal area with well-distributed rainfall the entire year and; therefore it is considered propitious to the transmission of gastrointestinal nematodes, mainly from the genus *Haemonchus*, due to the elevated environmental humidity. Each nematode species possesses some specific demands; however, the greatest survival occurs with medium temperatures, low radiation, low precipitation and high humidity (Rocha et al., 2007). Under high temperature and high humidity, L3s mostly migrate vertically to the tip of the canopy. Conversely, with a restriction on humidity, the larvae tend to remain in the feces or at the base of the plant (Silva et al., 2008; Santos et al., 2012). However L3 *H.contortus* are able to migrate across all the strata the summer season in humid subtropical climate (Gasparina et al., 2021).

When there was an increase in radiation, the animals from the Low and High Infection groups approached the dung, unlike the animals from the Medium Infection group, which always kept away. Humidity was always high, which favored the development of the parasitic cycle, even at high radiation. The non-significant effect of humidity in the models and the greater estimated ingestion of L3 verify that there was no L3 desiccation and that the more intense fecal odor, if present, did not stop the groups from approaching the contaminated dung. Radiation was shown to be determinant in the increase in foraging distance and influenced the migration of L3 in VRG pastures. Taking into consideration that in VRG the pasture height is very low when the animals exit the paddock, parasites may be exposed to the effects of the climate during the rest period, increasing the mortality of the free-living phases due to radiation and high temperatures. Thus, the grazing behavior of herbivorous mammals may also be affected by the interaction between humidity and radiation, the distribution of feces and parasites in the environment, and the physiological state of the animals (Hutchings et al., 2001b) and the growth of the pasture.

Understanding the infection risks in the different types of production systems is important for the environmental control of gastrointestinal nematodes, with a view to improving the management of pastures where the parasites are transmitted through the ingestion of L3 in the forage. The high prevalence of *Haemonchus* infections in this study had been expected, as it is the most prevalent parasite in the diverse regions of Brazil, mainly in the spring/summer (Amarante, 2014). The occurrence of parasites from the genus *Cooperia* had also been expected, due to the presence of cattle grazing in the area in the summer of 2015. Since *Haemonchus* is a parasite with a short life cycle mainly in the hot seasons, the feces avoidance phenomenon may become a way to control the ingestion of infective larvae. These feces avoidance strategies may be associated with other control interventions (Fox et al., 2013), such as a trade-off relationship between guaranteeing better nutrition or activating the

immune system (even partially), as in the case of the animals from the High and Medium Infection groups in this experiment. For instance, the results of this study demonstrated that the L3 ingestion control strategy for the High Infection group (i.e., keeping away from the dung) was caused by a discharge of the immune response each time the animals approached the feces in the pasture.

Finally, the distancing model, i.e. the distance that the lamb foraged in relation to the dung, indicated entry/exit, which is associated to radiation, larvae dissection and grazing system, IgG and mainly L3 ingestion influence on the final foraging distance. The ingestion of L3 from the pasture, in turn, was controlled by the level of infection, FEC and CV as demonstrated in the mixed model for L3.

## Conclusion

Crioula Lanada lambs increased the foraging distance from the dung after being naturally infected by gastrointestinal nematodes. However, this was not directly related to parasitic infection level, but to the dynamics of the solar radiation and parasitological and immunological factors in the VRG, which differed in time of discharge and persistence in animals with low, medium and high infection.
